# Changes in Prescribed Opioid Dosages Among Patients Receiving Medical Cannabis for Chronic Pain, New York State, 2017-2019

**DOI:** 10.1001/jamanetworkopen.2022.54573

**Published:** 2023-01-30

**Authors:** Trang Nguyen, Yunshu Li, Danielle Greene, Sharon Stancliff, Nicole Quackenbush

**Affiliations:** 1 New York State Department of Health, Albany; 2 Department of Epidemiology and Biostatistics, University at Albany School of Public Health, Rensselaer, New York; 3 City University of New York Graduate School of Public Health and Health Policy, New York, New York; 4 New York State Office of Cannabis Management, Albany

## Abstract

**Importance:**

Patients with chronic pain often receive long-term opioid therapy (LOT), which places them at risk of opioid use disorder and overdose. This presents the need for alternative or companion treatments; however, few studies on the association of medical cannabis (MC) with reducing opioid dosages exist.

**Objective:**

To assess changes in opioid dosages among patients receiving MC for longer duration compared with shorter duration.

**Design, Setting, and Participants:**

This cohort study of New York State Prescription Monitoring Program data from 2017 to 2019 included patients receiving MC for chronic pain while also receiving opioid treatment. Of these, patients receiving LOT prior to receiving MC were selected. Individuals were studied for 8 months after starting MC. Data were analyzed from November 2021 to February 2022.

**Exposures:**

Selected patients were divided into 2 groups based on the duration of receiving MC: the nonexposure group received MC for 30 days or fewer, and the exposure group received MC for more than 30 days.

**Main Outcomes and Measures:**

The main outcome was opioid dosage, measured by mean daily morphine milligram equivalent (MME). Analyses were conducted for 3 strata by opioid dosage prior to receiving MC: MME less than 50, MME of 50 to less than 90, and MME of 90 or greater.

**Results:**

A total of 8165 patients were included, with 4041 (median [IQR] age, 57 [47-65] years; 2376 [58.8%] female) in the exposure group and 4124 (median [IQR] age, 54 (44-62) years; 2370 [57.5%] female) in the nonexposure group. Median (IQR) baseline MMEs for the exposure vs nonexposure groups were 30.0 (20.0-40.0) vs 30.0 (20.0-40.0) in the lowest stratum, 60.0 (60.0-70.0) vs 60.0 (60.0-90.0) in the middle stratum, and 150.0 (100.0-216.2) vs 135.0 (100.0-218.0) in the highest stratum. During follow-up, significantly greater reductions in opioid dosage were observed among the exposure group. A dose-response association of patients’ opioid dosage at baseline was observed with the differences in the monthly MME reductions between exposure and nonexposure groups, with a difference of −1.52 (95% CI, −1.67 to −1.37) MME for the lowest stratum, −3.24 (95% CI, −3.61 to −2.87) MME for the middle stratum, and −9.33 (95% CI, −9.89 to −8.77) MME for the highest stratum. The daily MME for the last month of the follow-up period among patients receiving longer MC was reduced by 48% in the lowest stratum, 47% in the middle stratum, and 51% in the highest stratum compared with the baseline dosages. Among individuals in the nonexposure group, daily MME was reduced by only 4% in the lowest stratum, 9% in the middle stratum, and 14% in the highest stratum.

**Conclusions and Relevance:**

In this cohort study of patients receiving LOT, receiving MC for a longer duration was associated with reductions in opioid dosages, which may lower their risk of opioid-related morbidity and mortality.

## Key Points

**Question:**

Is receiving medical cannabis for a longer duration associated with reducing prescription opioid dosages among patients receiving long-term opioid therapy?

**Findings:**

In this cohort study among 8165 patients with chronic pain receiving long-term opioid therapy, receiving medical cannabis for a longer duration was associated with prescription opioid dosage reduction. Higher opioid dosages were associated with larger reductions.

**Meaning:**

These findings contribute evidence toward potential clinical benefits of medical cannabis in reducing prescription opioid intake, which may decrease patients’ risk of opioid overdose.

## Teaser

This cohort study assesses changes in prescribed opioid dosages among patients with chronic pain receiving medical cannabis for longer duration vs shorter duration.

## Introduction

The US has been experiencing an opioid crisis with multiple waves, starting with increased prescribing of opioids in the 1990s.^1^ Recently, opioid-involved overdose deaths in 2021 were the highest in US history, with more than 80 000 deaths.^2^ Opioid prescriptions decreased 47% by 2020, after a peak in 2012 with 255 million prescriptions.^3,4^ Despite significant reductions, many people are continuing to use opioids for chronic pain. In 2019, 1 in 5 adults had chronic pain^5^ and 22.1% of these had recently used prescription opioids for relief.^6^

National guidelines for prescribing opioids for chronic pain^7^ and states’ opioid regulations have resulted in discontinuation and reduced initiation of opioid prescriptions.^8^ Nonopioid medication options are limited for many individuals with pain; for example, nonsteroidal anti-inflammatory drugs (NSAIDs) are contraindicated for many patients experiencing chronic pain, particularly among older patients, who often experience a higher burden of pain.^9^ Without humane tapering of opioids and effective alternatives for patients receiving long-term opioid therapy (LOT), many of them are at high risk of overdose (through turning to the illicit market) and of suicide.^8,10^

Cannabis has gained popularity as a potential pain management modality.^11,12,13^ However, its role in reducing the need for prescription opioids or the dosage of opioids remains controversial due to limited research.^14^ Most studies relied on survey data or small samples of individuals who used cannabis in combination with opioids for pain management.^15^ Results showed individuals self-reported reduction in opioid use and improvement in pain management. A meta-analysis on the association of cannabinoids with reduced opioid use for analgesia found some support in observational studies but little evidence from clinical trials.^16^ Several trials used cannabis-derived products for a short time, which may not reflect how patients actually use cannabis as an alternative therapy.^16^

Medical cannabis (MC) programs present opportunities to investigate the potential therapeutic benefits of MC for pain management and its possible adjunctive use with opioid analgesics. Most MC programs regulate the licensure, cultivation, distribution, and sale of products and require patients to be authorized or certified by a health care practitioner to participate in the program. A 2021 study of patients receiving LOT in Canada by Lee et al^17^ found that MC authorization was associated with a significant weekly oral morphine equivalent reduction among individuals receiving high dosage of opioids (ie, oral morphine equivalent >100).^17^ However, among individuals receiving lower dosages, a weekly increase was observed. These findings provide direct evidence for the association between MC authorization and changes in opioid dosages. However, Lee et al^17^ measured exposure by authorization to receive MC rather than the actual receipt of MC and the length of exposure.

New York state implemented a MC program^18^ in January 2016 under the authority of the 2014 Compassionate Care Act.^19^ Chronic pain was added in regulation as a qualifying medical condition for receiving MC in March 2017.^20^ This study aims to assess the association between receiving MC for chronic pain and opioid dosage. Specifically, we examined the changes in opioid dosages among individuals receiving LOT who received MC for longer duration compared with shorter duration.

## Methods

### Study Design

This cohort study was approved by the New York State Department of Health’s institutional review board. This study used deidentified secondary data and individual patients’ identity could not be determined through the aggregated results. Per the Common Rule, informed consent was waived. The study was conducted by monitoring opioid dosage of 2 groups (receiving MC for a longer duration, ie, exposure group, and receiving MC for a shorter duration, ie, nonexposure group) for 240 days or 8 months (intervention period). This cohort study adhered to the Strengthening the Reporting of Observational Studies in Epidemiology (STROBE) reporting guideline for cohort studies.

### Data Source

The New York State Medical Cannabis Program (eAppendix 1 in Supplement 1) provides access to MC products to patients with qualifying medical conditions (eAppendix 2 in Supplement 1) who receive a certification from their health care practitioner and register with the program.^20^ No regulations prohibit the use of opioids and MC at the same time. The decision of appropriate therapy was at the discretion of the health care practitioner certifying the patient. The Medical Marijuana Data Management System (MMDMS) collects records of MC certifications, which include patients’ qualifying medical conditions. Records of dispensed MC and opioid prescriptions, reported to the New York state Prescription Monitoring Program registry (PMP), for these individuals were linked via unique identifiers.^21^

### Study Population

We examined MMDMS and PMP records from March 2017 through the end of 2019. We selected adults with at least 1 MC dispensed for chronic pain and 1 opioid prescription at the starting date of the first MC dispensing (index date). Patient were considered to be receiving LOT if they had a minimum of 120 cumulative days of opioid prescriptions or 10 or more prescriptions within the 12 months prior to the index date.^17^

### Exposure Definition

The exposure group included adults with more than 30 days of MC during the first 90-day intervention period. Patients in the nonexposure group received no more than 30 days of MC in the first 90 days and subsequently did not receive MC for the rest of the follow-up period.

### Outcome Definition

The study outcome, opioid dosage, was measured by 30-day (monthly) mean daily morphine milligram equivalent (MME). For each month, prescribed opioid amounts were used in combination with strength conversion factors to calculate the total MME. Subsequently, the total MME was divided by the supply days in that month to calculate the mean daily MME. Each patient was measured for 12 monthly MMEs during the preintervention period and 8 monthly MMEs for the intervention period.

### Covariates

The known factors associated with opioid dosages, such as age, sex, source of payment, and baseline MME prior to receiving MC, were included as covariates for analyses. Since payments for MC dispensing are typically not covered by third-party payers, we used the source of payment for the most recent opioid prescription prior to receiving MC to classify insurance type.

### Exclusion Criteria

Exclusion criteria were individuals aged younger than 18 years, identified as terminally ill on their MC certification, living out of New York state, received opioids for treatment of opioid use disorder, or received extremely high dosages of opioids (480 MME daily, sign of outliers or diversion),^22^ more than the NYS limit of 90 supply days,^23^ opioids that are not typically used in outpatient settings, cold formulations containing opioids, or opioids from veterinarians for animals. To ensure consistent exposure to MC,^24^ patients with a large gap (≥30 days) in receiving MC in the first 90 days were excluded.

### Statistical Analysis

We conducted χ^2^ and Fisher exact tests and examined medians and IQRs to check for equivalency in the distributions of age, sex, insurance type, and baseline MME between groups. Since baseline MME was shown to be associated with changes in follow-up opioid dosages,^17^ analyses were conducted for 3 strata of MME: less than 50, 50 to less than 90, and 90 or greater.

To adjust for confounders at baseline, a propensity score (PS) method was used to estimate the average treatment effect on treated (ATT) weights.^25,26,27^ For each stratum, the ATT weights were used in the PS-based controlled interrupted time series (CITS) model^27,28,29^ to assess the differences in the MME trends between the exposure and nonexposure groups during the preintervention period. The same models also provided the association between the length of receiving MC and opioid dosages after the intervention started. All results were set at a statistical significance level of 2-sided *P* = .05. Statistical analyses were conducted using SAS software version 9.4 (SAS Institute). Data were analyzed from November 2021 to February 2022.

## Results

**Figure 1.**
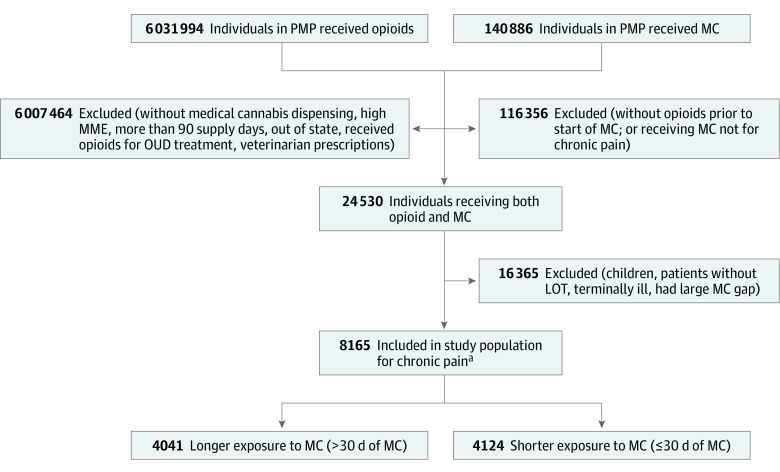
Study Population From the New York State Prescription Monitoring Program (PMP) Registry, 2017-2019 MME indicates morphine milligram equivalent; OUD, opioid use disorder. ^a^Patients receiving long-term opioid therapy (LOT; ≥120 cumulative days of opioid prescriptions or ≥10 or more opioid prescriptions during the year prior to medical cannabis [MC] start date) that were certified and received MC for chronic pain.

Figure 1 shows the selection of the study population. Between 2017 and 2019, there were 8165 patients receiving LOT who also received MC for chronic pain. Of those, 4041 patients (median [IQR] age, 57 [47-65] years; 2376 [58.8%] female) received MC for a longer duration and 4124 patients (median [IQR] age, 54 [44-62] years; 2370 [57.5%] female) received MC for a shorter duration.

Table 1 provides distributions for age, sex, insurance, and MME at baseline for all patients and stratified by baseline MME. Approximately half of all patients were prescribed less than 50 MME, a third were prescribed 90 MME or greater, and the remainder were receiving 50 to less than 90 MME at baseline. Overall, there were statistically significant differences in the distributions of age and source of payments between the exposure and nonexposure groups. (Table 1) The exposure group included more patients aged 65 years and older (1083 patients [26.8%] vs 845 patients [20.5%]), and fewer patients aged 18 to 44 years (827 patients [20.5%] vs 1063 patients [25.8%]) than the nonexposure group. More than half of patients in each group had private insurance (2548 patients [63.1%] in the exposure group and 2542 patients [61.6%] in the nonexposure group). The exposure group had fewer patients with Medicaid insurance (123 patients [3.0%] vs 230 patients [5.6%]) than the nonexposure group. Daily MME at baseline was similar for exposure and nonexposure groups (Table 1). There was no missing information for these variables.

**Table 1.** Characteristics of Individuals With Chronic Pain by Baseline MME and Duration of MC Exposure, New York State, 2017-2019

Characteristic	Overall			MME <50			MME 50 to <90			MME ≥90		
	MC use, No. (%)		*P* value	MC use, No. (%)		*P* value	MC Use, No. (%)		*P* value	MC use, No. (%)		*P* value
	Longer (n = 4041)	Shorter (n = 4124)		Longer (n = 2009)	Shorter (n = 2002)		Longer (n = 701)	Shorter (n = 757)		Longer (n = 1331)	Shorter (n = 1365)	
Age, y												
Median (IQR)	57 (47-65)	54 (44-62)	NA	58 (47-67)	54 (44-64)	NA	56 (47-65)	54 (46-62)	NA	56 (46-63)	53 (43-60)	NA
18-44	827 (20.5)	1063 (25.8)	<.001^a^	402 (20.0)	529 (26.4)	<.001^a^	139 (19.8)	167 (22.1)	.11^a^	286 (21.5)	367 (26.9)	.001^a^
45-64	2131 (52.7)	2216 (53.7)		994 (49.5)	1014 (50.7)		380 (54.2)	428 (56.5)		757 (56.9)	774 (56.7)	
≥65	1083 (26.8)	845 (20.5)		613 (30.5)	459 (22.9)		182 (26.0)	162 (21.4)		288 (21.6)	224 (16.4)	
Sex												
Male	1665 (41.2)	1754 (42.5)	.22^a^	786 (39.1)	773 (38.6)	.74^a^	280 (39.9)	324 (42.8)	.27^a^	599 (45.0)	657 (48.1)	.10^a^
Female	2376 (58.8)	2370 (57.5)		1223 (60.9)	1229 (61.4)		421 (60.1)	433 (57.2)		732 (55.0)	708 (51.9)	
Payment												
Medicare	878 (21.7)	870 (21.1)	<.001^a^	464 (23.1)	416 (20.8)	<.001^b^	149 (21.3)	166 (21.9)	.11^b^	265 (19.9)	288 (21.1)	.004^b^
Medicaid	123 (3.0)	230 (5.6)		76 (3.8)	136 (6.8)		20 (2.8)	35 (4.6)		27 (2.0)	59 (4.3)	
Private insurance	2548 (63.1)	2542 (61.6)		1220 (60.7)	1219 (60.9)		462 (65.9)	463 (61.2)		866 (65.1)	860 (63.0)	
Others	492 (12.2)	482 (11.7)		249 (12.4)	231 (11.5)		70 (10.0)	93 (12.3)		173 (13.0)	158 (11.6)	
Daily MME at baseline, median (IQR)	50.0 (30.0-97.5)	55.3 (30.0-98.5)	NA	30.0 (20.0-40.0)	30.0 (20.0-40.0)	NA	60.0 (60.0-70.0)	60.0 (60.0-69.0)	NA	150.0 (100.0-216.2)	135.0 (100.0-218.0)	NA

Abbreviations: MC, medical cannabis; MME, morphine milligram equivalent; NA, not applicable.

^a^ χ^2^ Test.

^b^ Fisher exact test.

**Figure 2.**
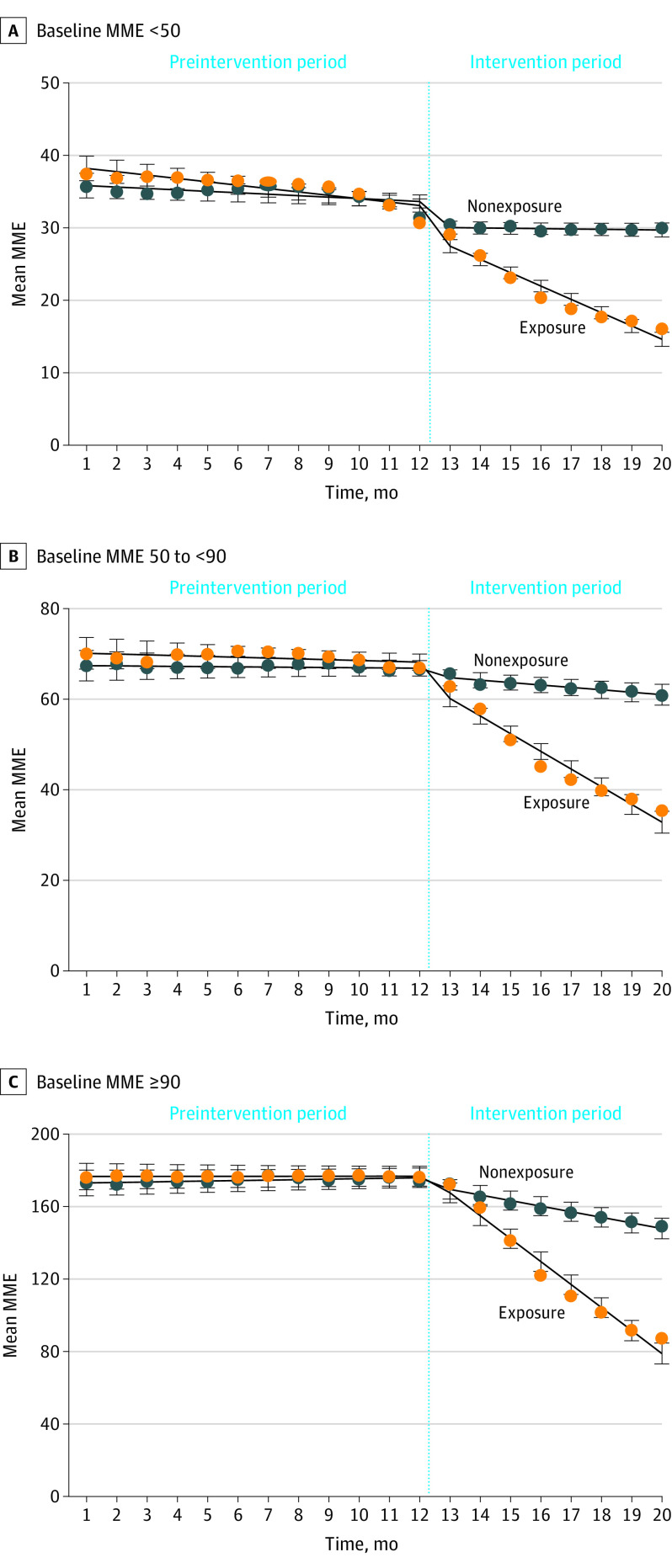
Observed and Model Estimated 30-Day Average Daily Morphine Milligram Equivalent (MME) Stratified by Baseline MME, New York State, 2017-2019 The exposure group was patients who received medical cannabis for more than 30 days, and the nonexposure group was patients who received medical cannabis for 30 days or fewer. Estimates are based on the propensity score–based weighted control interrupted time series models, adjusted for age, sex, and source of payment. Whiskers indicate 95% CI.

Figure 2 shows the observed and model-estimated 30-day mean daily MME trends for the exposure and nonexposure groups, stratified by baseline MME. The mean MMEs for the exposure group generally started higher than the nonexposure group across strata. In the 2 strata with lower MME (Figure 2A and B), both groups showed small downward trends during the preintervention period. However, statistically significant trends were found only in the lowest MME stratum: a −0.20 (95% CI, −0.31 to −0.09) MME monthly reduction was observed in the nonexposure group, while the exposure group experienced a −0.27 (−0.43 to −0.11) MME reduction (Table 2). Before the intervention started, no significant MME changes were observed in either group for patients with a baseline dosage of 50 MME or greater.

**Table 2.** Monthly Mean Daily MME for Individuals With Chronic Pain Receiving Medical Cannabis for Longer vs Shorter Duration, by Baseline MME, New York State, 2017-2019^a^

Measure	MME <50	MME 50 to <90	MME ≥90
Preintervention period (95% CI)			
Starting MME for nonexposure	36.02 (34.23 to 37.82)	67.43 (63.84 to 71.01)	172.74 (165.34 to 180.14)
Difference in starting MME^b^	2.64 (0.11 to 5.18)	2.88 (−2.29 to 8.05)	3.82 (−6.71 to 14.36)
Daily MME trend for nonexposure group each 30 d	−0.20 (−0.31 to −0.09)	−0.05 (−0.31 to 0.21)	0.26 (−0.13 to 0.66)
Difference in daily MME trend each 30 d^b^	−0.27 (−0.43 to −0.11)	−0.13 (−0.51 to 0.25)	−0.25 (−0.81 to 0.32)
Intervention period difference (95% CI)^b^			
Daily MME trends each 30 d	−1.52 (−1.67 to −1.37)	−3.24 (−3.61 to −2.87)	−9.33 (−9.89 to −8.77)
Total MME over full intervention period	−14.53 (−17.45 to −11.61)	−29.49 (−35.94 to −23.04)	−69.81 (−87.09 to −52.53)

Abbreviation: MME, morphine milligram equivalent.

^a^ Estimates based on the propensity score–based weighted control interrupted time series models, adjusted for age, sex, and source of payment. The exposure group was patients who received medical cannabis for more than 30 days, and the nonexposure group was patients who received medical cannabis for 30 days or fewer.

^b^ Differences calculated as exposure − nonexposure.

At baseline, the observed mean (SD) MMEs for the exposure vs nonexposure groups were 30.7 (17.3) vs 31.4 (18.5) in the lowest stratum, 66.9 (19.9) vs 66.6 (17.9) for the middle stratum, and 176.2 (106.9) vs 174.0 (106.1) for the highest stratum (Figure 2). After the intervention started, larger reductions in the daily MME were observed in the exposure group compared with the nonexposure group (Figure 2).

Among the lowest MME stratum (Figure 2A), mean (SD) daily MME at the end of the intervention period for the exposure group was 16.0 (22.2), a 48% reduction from 30.7 MME at the baseline, compared with 30.0 (25.4) for the nonexposure group, a 4% reduction from a baseline MME of 31.4. The adjusted analyses found the exposure group had a larger monthly MME reduction compared with the nonexposure group, with a difference of −1.52 (95% CI, −1.67 to −1.37) MME (Table 2). This resulted in a total MME net reduction of −14.53 (95% CI, −17.45 to −11.61) MME over 8 months for the exposure group compared with the MME changes in the nonexposure group.

Among patients with a baseline MME of 50 to less than 90 (Figure 2B), the mean (SD) daily MME at the end of the intervention period was 35.4 (37.6) for the exposure group (a 47% reduction) vs 60.8 (35.7) for the nonexposure group (a 9% reduction). The adjusted analysis showed a significant difference of −3.24 (95% CI, −3.61 to −2.87) MME in monthly MME reduction between the exposure group and the nonexposure group (Table 2). After 8 months, a total MME reduction of −29.49 (95% CI, −35.94 to −23.04) MME was observed for the exposure group compared with the nonexposure group.

For adults with a baseline MME of 90 or greater, by the end of the intervention period, the mean (SD) daily MME was 87.2 (106.6) for the exposure group (a 51% reduction from 176.2 MME at the baseline) vs 149.0 (114.0) for the nonexposure group (a 14% reduction from 174 MME at baseline) (Figure 2C). The adjusted analyses showed a monthly reduction difference of −9.33 (95% CI, −9.89 to −8.77) MME between groups. A large MME net reduction of −69.81 (95% CI, −87.09 to −52.53) was observed for the exposure group over the 8-month intervention.

**Figure 3.**
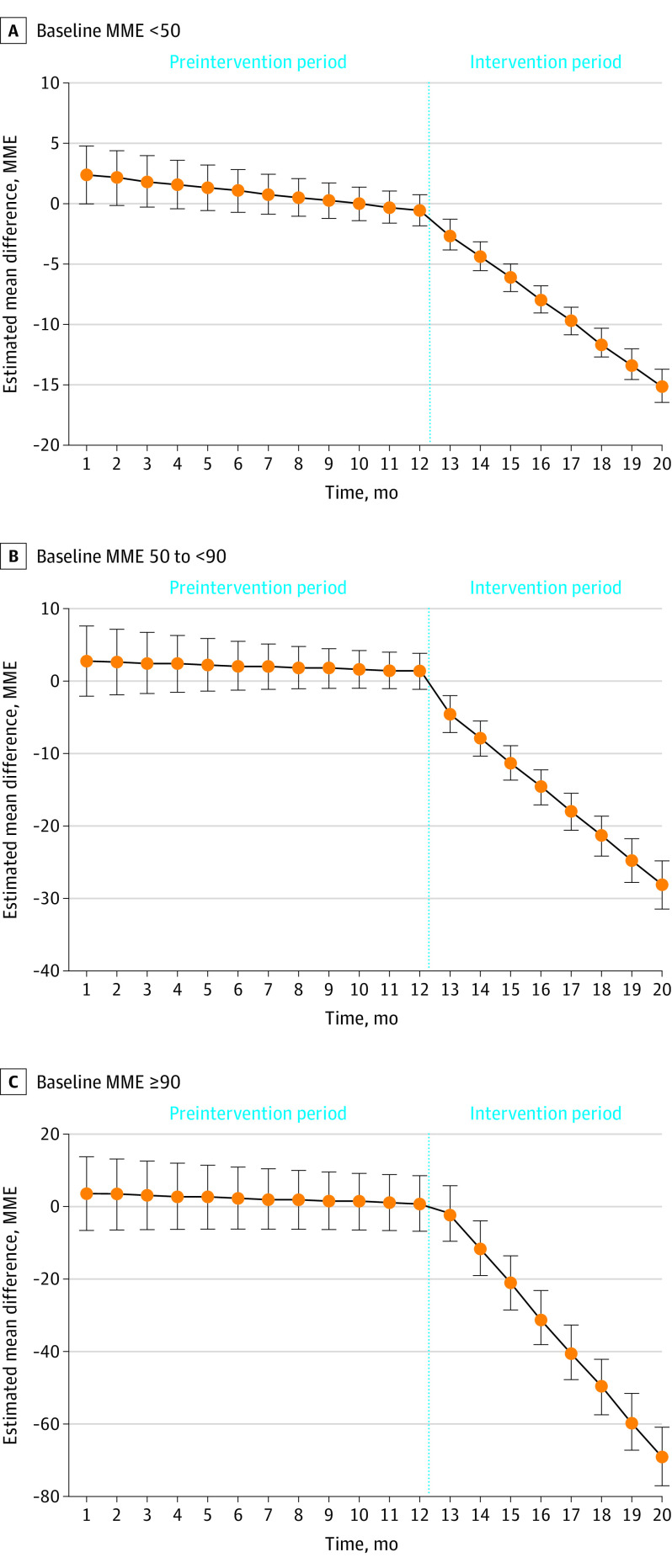
Estimated Differences for Monthly Mean Daily Morphine Milligram Equivalent (MME) Between Exposure and Nonexposure Groups by Baseline MME, New York State, 2017-2019 The exposure group was patients who received medical cannabis for more than 30 days, and the nonexposure group was patients who received medical cannabis for 30 days or fewer. Estimates are based on the propensity score–based weighted control interrupted time series models, adjusted for age, sex, and source of payment. Whiskers indicate 95% CIs.

Additional analyses for daily MME differences between the exposure and nonexposure groups found no statistically significant differences before the intervention. After the intervention started, the differences between groups significantly widened over time (Figure 3).

## Discussion

This cohort study of patients in New York state receiving LOT found that receiving MC for chronic pain for a longer duration was associated with significant reductions in opioid dosages. Larger reductions were observed for patients receiving higher opioid dosages at the baseline. Among patients with a baseline dosage of less than 50 MME, a significant monthly reduction in the daily opioid dosages (−1.52 MME) was observed among adults receiving long-term MC compared with those with a shorter MC duration. This reduction was 5.6 times larger than the difference between groups prior to receiving MC (−0.27 MME). The higher the patients’ opioid dosage at baseline, the larger the monthly reduction: −3.24 MME for patients receiving 50 to less than 90 MME and −9.33 MME for patients receiving 90 MME or greater. These reductions were 24.9 times larger in the middle stratum and 37.3 times larger in the highest stratum than the differences prior to receiving MC.

Among patients receiving MC for longer, the mean daily dosage for the group with baseline dosage of 90 MME or greater was reduced to less than 90 MME (87.2), and the mean dosage among those in the 50 to less than 90 MME stratum was reduced to less than 50 MME (35.4 MME). If replicable, such reductions might impact future morbidity and mortality of this population.

In the month immediately after the intervention started, both groups receiving MC experienced a reduction in prescribed opioid dosages; patients in the nonexposure group stopped receiving MC after a maximum of 30 supply days, while patients in the exposure group continued to receive MC longer. This could explain the larger and sharper reductions in MME for adults with longer MC. Even after taking the reductions among adults with shorter MC into the calculation, the estimated total MME net reductions for patients with longer MC over the 8-month intervention were 14.53 MME in the lowest baseline stratum, 29.49 in the middle baseline stratum, and 69.81 in the highest baseline stratum. Among patients receiving MC for longer, daily MME was reduced by 47% to 51% of the baseline dosages after the 8-month intervention. Adults receiving MC for less time reduced their baseline dosages by just 4% to 14%.

We did not have information to determine whether patients chose MC as an adjunct therapy to decrease opioid dosages or in response to clinician pressure to decrease opioid dosages. However, no regulations prohibited the use of opioids and MC simultaneously. The clinical significance of the opioid reductions associated with MC is illustrated by the Centers for Disease Control and Prevention guideline on prescribing opioids for chronic pain,^7^ which highlights the escalation of overdose risk as prescribed MME increases. Clinicians caring for patients receiving LOT should discuss the role MC may play in pain management and information on risks and benefits. When patients wish to use MC and their current clinician is unable or unwilling to certify patients, they should be referred to practitioner who could certify patients for MC.

Patients with chronic pain who are insured by Medicaid receive opioids at a higher rate than privately insured individuals and are more likely to be receiving LOT.^30^ Few patients in the study population were insured by Medicaid, and of those, many who initiated MC did not continue. This may be because MC is typically not covered by insurance and was thus unaffordable. The lack of affordability of a medication that may reduce the need for opioids for pain management and perhaps risk of opioid use disorder and overdose is a health inequity deserving further examination.

MC is documented to have side effects: among the most common are psychoactive effects, dizziness, and dry mouth. Other concerns include cannabis use disorder and withdrawal symptoms.^31^ New York state regulations allowed MC products with THC to be sold by dispensaries; however, THC was limited at a low dose of 10 mg. Therefore, the risk of adverse events and cannabis use disorder is low if patients use MC as directed. A previous study of MC found that while 37.3% of patients experienced 1 or more adverse events, most were mild, and less than 2% of patients required dose adjustment or cessation, suggesting that MC is far safer than opioids.^32^ However, caution is still urged in certifying patients with some comorbidities, including coronary artery disease, arrhythmias, or a history of psychosis, or patients who are pregnant.^33^

This study had several strengths. To our knowledge, this is the first population-based study with a large sample size that evaluated the association between the length of receiving MC and reduction in daily opioid dosages. The length of receiving MC was used to rigorously measure exposure status and concurrent opioids. Using the CITS model methods on patient-level data allowed us to assess preintervention trends and generate the net differences in the daily MME between exposure and nonexposure groups after the intervention started. Even though not all confounders were controlled for, it is highly unlikely that the greater and sharper reductions right after the intervention started and the continuing reductions in the exposure groups are due to factors other than the receiving MC for longer in patients from the 3 baseline MME levels. Future studies should further examine the specific effects of different types or combinations of MC products and dosages on prescribed opioid dosages.

### Limitations

This study has some limitations. First, our study included no assessment of MC dosages, product type, or ratio of THC to CBD among products received to determine whether there would be any difference by these variables. However, we were able to confirm receipt of MC and used the lengths of MC supply to measure exposure. This is an observational study, which could be subject to selection bias. However, as patients in both exposure and nonexposure groups actively sought and initially received MC, this could only impact the generalizability of the results. There was a lack of race and ethnicity information and clinical information, such as comorbidities or the causes of chronic pain. However, exposure and nonexposure groups in each stratum were comparable regarding several known confounders. Furthermore, the PS-based CITS models (used to control for confounders and selection bias) are advanced techniques to improve the validity and accuracy, such that the results may comparable with randomized clinical trial results.^34,35^ We do not know whether any patients who stopped using prescribed opioids switched to illicit opioids or whether those who stopped using MC switched to the unregulated market. Additionally, we did not have data on patients’ pain level; therefore, we were unable to assess pain management improvement.

## Conclusions

This cohort study found that receiving MC for longer was associated with opioid dosage reductions. The reductions were larger among individuals who were prescribed higher dosages of opioids at baseline. These findings contribute robust evidence for clinicians regarding the potential benefits of MC in reducing the opioid burden for patients receiving LOT and possibly reduce their risk for overdose. Further research is needed to confirm the causal effect of MC and conduct benefit-risk assessment of MC as opioid alternative or companion treatments to address management of chronic pain and the opioid crisis.

## Notes

Supplement 1. **eAppendix 1.** Summary Information About the New York State Medical Cannabis (Previously Medical Marijuana) Program, January 2016-December 2019 **eAppendix 2.** Qualifying Conditions for Medical Cannabis Certification as of March 2017

Supplement 2.
Data Sharing Statement
