# Supplementary material for: Changes in Prescribed Opioid Dosages Among Patients Receiving Medical Cannabis for Chronic Pain, New York State, 2017-2019
Source: JAMA Netw Open. 2023 Jan 30;6(1):e2254573. doi: 10.1001/jamanetworkopen.2022.54573 (PMC9887491; doi:10.1001/jamanetworkopen.2022.54573)
Supplement: Supplement 1. — eAppendix 1. Summary Information About the New York State Medical Cannabis (Previously Medical Marijuana) Program, January 2016-December 2019 eAppendix 2. Qualifying Conditions for Medical Cannabis Certification as of March 2017 [file jamanetwopen-e2254573-s001.pdf]

# Supplemental Online Content

Nguyen T, Li Y, Greene D, Stancliff S, Quackenbush N. Changes in prescribed opioid dosages among patients receiving medical cannabis for chronic pain, New York state, 2017-2019. *JAMA Netw Open*. 2023;6(1):e2254573. doi:10.1001/jamanetworkopen.2022.54573

**eAppendix 1.** Summary Information About the New York State Medical Cannabis (Previously Medical Marijuana) Program, January 2016-December 2019

**eAppendix 2.** Qualifying Conditions for Medical Cannabis Certification as of March 2017

This supplemental material has been provided by the authors to give readers additional information about their work.

© 2023 Nguyen T et al. *JAMA Network Open.*

**eAppendix 1.** Summary Information About the New York State Medical Cannabis (Previously Medical Marijuana) Program, January 2016-December 2019

Starting in January 2016, NYS residents with qualifying medical conditions (Supplement 2) could be certified by a healthcare provider to participate in the medical cannabis program in New York State. At the time of the data used for this study, health care providers such as physicians, nurse practitioners, and physician assistants, who were licensed, registered, or certified by New York State (as appropriate to their profession), had to complete at minimum a two-hour course approved by New York State. The course requirements for practitioners included the following content: the pharmacology of cannabis; contraindications; side effects; adverse reactions; overdose prevention; drug interactions; dosing; routes of administration; risks and benefits; warnings and precautions; and abuse and dependence. After completing the course and registering with the NYS Medical Cannabis Program, healthcare providers could certify patients to receive medical cannabis from licensed and registered dispensaries.

Patients were encouraged to first communicate with their treating practitioners to see if medical cannabis may be appropriate for their condition. For those patients whose practitioners chose not to participate in the medical cannabis program, a list of existing providers who consented to have their information publicly available was presented on the program website.

There were three steps for patients to become certified for medical cannabis and purchase medical cannabis products during the study period. First, patients had to be certified by the registered health care providers (described above). During the certification process, providers had to indicate patient's qualifying medical condition for medical cannabis and whether patient was terminally ill; could make recommendations for medical cannabis products and dosing; and specify the expiration date on the certification. Each certification could be issued up to one year. Providers used New York State's Medical Cannabis Data Management System (MCDMS). After patients were certified for medical cannabis, they were provided a medical cannabis certification by their provider. Providers could edit or cancel an active certification. Second, patients were required to register online to receive a registry ID card. Third, patients could purchase medical cannabis by visiting a registered organization's medical cannabis dispensary to purchase product with their registry ID card and certification. At the time of this study, the total amount of product that may be dispensed by a registered organization could not exceed a thirty-day supply at a time. Information for medical cannabis products dispensed by these facilities was required to be submitted to the New York State Prescription Monitoring Program Registry.

The product forms available to medical cannabis patients for purchase at registered organizations were limited to those forms approved by the NYSDOH during the study time period. Registered organizations were permitted to manufacture medical cannabis products in forms approved by the NYSDOH. The NYSDOH could approve additional forms and routes of administration. Each registered organizations could produce their own brands of medical cannabis products, with prior approval of the NYSDOH and were required to have at least one brand that has a low tetrahydrocannabinol (THC) content and high cannabidiol (CBD) content, and at least one brand with approximately equal amounts of THC and CBD. Products containing THC were limited to 10mg per dose. Some examples of available medical cannabis products available during the

© 2023 Nguyen T et al. *JAMA Network Open.*

study period were vape cartridge/pen, capsule/tablets, tincture, oral spray, oral powder, lozenges, and metered ground plant preparation for vaporization. Products from the dispensaries were held to a high-quality standard with limitations on the types of excipients that could be added to products, specific packaging and labeling requirements, and regularly tested by independent laboratories that were certified by the NYS Department of Health’s Environmental Laboratory Approval Program for cannabinoid content and contaminants.

Substance use disorder (SUD), including opioid use disorder (OUD), became a qualifying condition for receiving medical cannabis starting September 2018. However, we only selected patients with chronic pain as a qualifying medical condition for receiving medical cannabis for this study. Furthermore, based on the prescription data, we excluded patients who received buprenorphine prescriptions for OUD treatment.

There was no regulation that prohibited the use of opioids and medical cannabis at the same time. The decision of appropriate therapy was at the discretion of the practitioner certifying the patient.

© 2023 Nguyen T et al. *JAMA Network Open.*

**eAppendix 2.** Qualifying Conditions for Medical Cannabis Certification as of March 2017

1. Cancer,
2. Positive status for human immunodeficiency virus or acquired immune deficiency syndrome, provided that the practitioner has obtained from the patient consent for disclosure of this information that meets the requirements set forth in sections twenty-seven hundred eighty and twenty-seven hundred eighty-two of the public health law;
3. Amyotrophic lateral sclerosis (ALS),
4. Parkinson's disease,
5. Multiple sclerosis,
6. Spinal cord injury with spasticity,
7. Epilepsy,
8. Inflammatory bowel disease,
9. Neuropathy,
10. Huntington's disease, and
11. Any severe debilitating pain that the practitioner determines degrades health and functional capability; where the patient has contraindications, has experienced intolerable side effects, or has experienced failure of one or more previously tried therapeutic options; and where there is documented medical evidence of such pain having lasted three months or more beyond onset, or the practitioner reasonably anticipates such pain to last three months or more beyond onset; ***Defined as "chronic pain"***

© 2023 Nguyen T et al. *JAMA Network Open.*
