# Supplementary material for: Changes in Prescribed Opioid Dosages Among Patients Receiving Medical Cannabis for Chronic Pain, New York State, 2017-2019
Source: JAMA Netw Open. 2023 Jan 30;6(1):e2254573. doi: 10.1001/jamanetworkopen.2022.54573 (PMC9887491; doi:10.1001/jamanetworkopen.2022.54573)
Supplement: Supplement 2. — Data Sharing Statement [file jamanetwopen-e2254573-s002.pdf]

# Data Sharing Statement

Nguyen. Changes in Prescribed Opioid Dosages Among Patients Receiving Medical Cannabis for Chronic Pain, New York State, 2017-2019. *JAMA Netw Open*. Published January 30, 2023. doi:10.1001/jamanetworkopen.2022.54573

**Data**
**Data available:** No
